# Supplementary material for: Regional Living Conditions and Individual Dietary Characteristics of the Russian Population
Source: Nutrients. 2023 Jan 12;15(2):396. doi: 10.3390/nu15020396 (PMC9862910; doi:10.3390/nu15020396)
Supplement: Supplementary file 1 [file nutrients-15-00396-s001.zip › nutrients-2139319-supplementary.pdf]

Supplementary Table S1. Association of individual and regional characteristics with dietary patterns in women (n = 11,240).

| Characteristics | | Prudent DP | | Salt-rich DP | | Meat-based DP | | Mixed DP | | Cardioprotective DP | |
|---|---|---|---|---|---|---|---|---|---|---|---|
| | | B-coeff. | *p*-value | B-coeff. | *p*-value | B-coeff. | *p*-value | B-coeff. | *p*-value | OR | 95% CI |
| Individual characteristics | | | | | | | | | | | |
| Place of residence (reference: urban) | | 0.037 | 0.43 | 0.027 | 0.50 | -0.040 | 0.61 | 0.003 | 0.93 | 0.75 | 0.60-0.94 |
| Has a family (reference: none) | | 0.055 | 0.009 | 0.095 | <0.001 | 0.169 | <0.001 | -0.002 | 0.93 | 1.04 | 0.91-1.19 |
| Education, (reference: other than higher) | | 0.122 | <0.001 | -0.183 | <0.001 | 0.038 | 0.23 | 0.018 | 0.60 | 1.69 | 1.366-2.11 |
| Employment (reference: unemployed) | | 0.018 | 0.38 | 0.110 | <0.001 | 0.095 | <0.001 | -0.039 | 0.11 | 1.01 | 0.85-1.20 |
| Gastrointestinal diseases (reference: none) | | 0.058 | 0.032 | -0.073 | 0.002 | 0.022 | 0.58 | -0.091 | 0.002 | 0.96 | 0.82-1.11 |
| Peptic ulcer disease (reference: none) | | -0.032 | 0.47 | -0.007 | 0.85 | 0.045 | 0.11 | -0.011 | 0.69 | 0.84 | 0.65-1.09 |
| Diabetes mellitus (reference: none) | | -0.516 | <0.001 | -0.336 | <0.001 | 0.268 | <0.001 | 0.125 | 0.033 | 1.78 | 1.36-2.33 |
| Smoking (reference: never) | Quit | -0.144 | <0.001 | -0.088 | 0.009 | 0.021 | 0.57 | -0.084 | 0.004 | 0.98 | 0.82-1.17 |
| | Smoker | -0.383 | <0.001 | 0.143 | <0.001 | 0.075 | <0.001 | -0.135 | <0.001 | 0.75 | 0.61-0.92 |
| Age | | 0.001 | 0.41 | -0.009 | <0.001 | -0.002 | 0.042 | 0.008 | <0.001 | 1.01 | 1.00-1.02 |
| Body mass index | | -0.006 | <0.001 | 0.005 | 0.003 | 0.012 | <0.001 | 0.003 | 0.34 | 1.01 | 0.99-1.02 |
| Regional indices | | | | | | | | | | | |
| Socio-geographical index | | 0.131 | 0.002 | 0.011 | 0.76 | 0.094 | 0.052 | -0.065 | 0.14 | 1.25 | 1.10-1.42 |
| Demographic index | | 0.049 | 0.15 | 0.036 | 0.34 | 0.053 | 0.23 | -0.045 | 0.35 | 1.42 | 1.31-1.55 |
| Industrial index | | 0.028 | 0.38 | -0.027 | 0.33 | 0.068 | 0.094 | -0.041 | 0.45 | 1.07 | 1.01-1.14 |
| Mixed index | | -0.062 | <0.001 | -0.011 | 0.39 | 0.017 | 0.31 | 0.101 | <0.001 | 1.15 | 1.13-1.18 |
| Economic index | | -0.006 | 0.89 | 0.002 | 0.96 | 0.089 | 0.12 | -0.003 | 0.96 | 0.89 | 0.80-0.99 |

Supplementary Table S2. Association of individual and regional characteristics with dietary patterns in men (n = 6,814).

| Characteristics | | Prudent DP | | Salt-rich DP | | Meat-based DP | | Mixed DP | | Cardioprotective DP | |
|---|---|---|---|---|---|---|---|---|---|---|---|
| | | B-coeff. | *p*-value | B-coeff. | *p*-value | B-coeff. | *p*-value | B-coeff. | *p*-value | OR | 95% CI |
| Individual characteristics | | | | | | | | | | | |
| Place of residence (reference: urban) | | -0.041 | 0.54 | 0.120 | 0.017 | -0.068 | 0.12 | -0.080 | 0.10 | 0.69 | 0.46-1.04 |
| Has a family (reference: none) | | 0.054 | 0.032 | 0.010 | 0.60 | 0.091 | 0.018 | 0.041 | 0.29 | 0.83 | 0.66-1.04 |
| Education, (reference: other than higher) | | 0.112 | <0.001 | -0.135 | <0.001 | 0.042 | 0.17 | -0.013 | 0.82 | 1.73 | 1.28-2.34 |
| Employment (reference: unemployed) | | 0.076 | 0.068 | 0.069 | 0.051 | 0.127 | <0.001 | -0.029 | 0.46 | 1.05 | 0.75-1.46 |
| Gastrointestinal diseases (reference: none) | | 0.145 | <0.001 | -0.051 | 0.12 | 0.006 | 0.86 | 0.001 | 0.99 | 1.21 | 0.99-1.47 |
| Peptic ulcer disease (reference: none) | | -0.009 | 0.84 | 0.041 | 0.28 | 0.031 | 0.31 | -0.055 | 0.18 | 0.75 | 0.50-1.14 |
| Diabetes mellitus (reference: none) | | -0.484 | <0.001 | -0.429 | <0.001 | 0.065 | 0.41 | 0.297 | <0.001 | 2.23 | 1.28-3.90 |
| Smoking (reference: never) | Quit | -0.100 | <0.001 | 0.087 | <0.001 | 0.038 | 0.46 | -0.045 | 0.20 | 1.11 | 0.85-1.45 |
| | Smoker | -0.273 | <0.001 | 0.198 | <0.001 | 0.114 | 0.005 | -0.129 | 0.003 | 0.59 | 0.43-0.80 |
| Age | | 0.003 | 0.068 | -0.010 | <0.001 | 0.01 | 0.44 | 0.007 | <0.001 | 1.02 | 1.01-1.03 |
| Body mass index | | -0.010 | <0.001 | 0.004 | 0.073 | 0.012 | <0.001 | -0.001 | 0.99 | 1.01 | 0.99-1.03 |
| Regional indices | | | | | | | | | | | |
| Socio-geographical index | | 0.031 | 0.67 | -0.033 | 0.35 | 0.114 | 0.063 | -0.069 | 0.23 | 1.18 | 0.99-1.41 |
| Demographic index | | 0.015 | 0.81 | 0.055 | 0.068 | 0.012 | 0.85 | -0.083 | 0.31 | 1.31 | 1.02-1.67 |
| Industrial index | | 0.022 | 0.58 | -0.021 | 0.32 | 0.045 | 0.25 | -0.002 | 0.97 | 1.13 | 0.96-1.32 |
| Mixed index | | -0.046 | <0.001 | -0.039 | <0.001 | 0.016 | 0.26 | 0.060 | 0.003 | 1.15 | 1.07-1.24 |
| Economic index | | 0.024 | 0.68 | 0.020 | 0.40 | 0.060 | 0.37 | -0.058 | 0.45 | 0.91 | 0.73-1.13 |

Supplementary Table S3. Associations of individual and regional characteristics with a three-component cardioprotective dietary pattern (n = 18,054).

| **Characteristics** | | **OR** | **95% CI** |
|---|---|---|---|
| Individual characteristics | | | |
| Gender (reference: women) | | 0.65 | 0.56-0.75 |
| Place of residence (reference: urban) | | 0.86 | 0.74-0.99 |
| Has a family (reference: none) | | 1.05 | 1.01-1.10 |
| Education, (reference: other than higher) | | 1.42 | 1.23-1.64 |
| Employment (reference: unemployed) | | 1.12 | 1.03-1.23 |
| Gastrointestinal diseases (reference: none) | | 1.09 | 0.98-1.19 |
| Peptic ulcer disease (reference: none) | | 0.96 | 0.86-1.06 |
| Diabetes mellitus (reference: none) | | 1.65 | 1.48-1.83 |
| Smoking (reference: never) | Quit | 0.94 | 0.86-1.03 |
| | Smoker | 0.78 | 0.70-0.86 |
| Age | | 1.01 | 1.01-1.02 |
| Body mass index | | 1.01 | 1.01-1.02 |
| Regional indices | | | |
| Socio-geographical index | | 1.27 | 1.10-1.47 |
| Demographic index | | 1.16 | 1.03-1.31 |
| Industrial index | | 1.09 | 0.96-1.24 |
| Mixed index | | 1.16 | 1.10-1.22 |
| Economic index | | 1.04 | 0.94-1.16 |
